# Miasm and Fevers

**Published:** 1880-02

**Authors:** 


### MIASM AND FEVERS.

Abundant experience has already established the following facts regarding the appearance of intermittent fevers and the causes which are designated as *malaria:* First, that the real cause is to be sought for in the soil, where it is to be developed in greater intensity under favorable conditions of heat and warmth; second, that this poisonous substance, when the surface is dry, is lifted up a little above the surface by ascending currents, and can then be carried further or raised to a greater height by stronger draughts of air; third, that this substance, the cause of the malaria, is not developed in every soil of the same composition and the same degree of moisture, a circumstance which has repeatedly led to the assumption that it possesses the nature of a specific organism, which requires for its development not only the most favorable conditions, but first of all a *germ* from which it is developed.

From time immemorial, the Roman campagna has been known as one of the poisoned plague spots of the earth—hence the interest that naturally attaches to the investigations made there last spring by Klebs and Tommassi-Crudeli.

The malarial powers of different kinds of soil, of water, and of air, were tested. The solid and liquid portions of the former were tested separately. Under the supposition that the germs of the disease were organism, substances rich in infective matter were exposed to those conditions which have been found, by experience, most favorable to the development of the disease (30° to 40° C., or 86° to 104° F.; plenty of moisture deeper in the soil and rapid evaporation on the surface). Small particles of substances thus prepared, were transferred to different liquids for cultivation, and then experiments were made to determine whether, after frequent successive fractional cultivation, the same activity was present as in the substance first employed. Finally, the liquid was mechanically separated from the solid microscopic particles in the cultivated liquids, as in the original, by filtration through gypsum and other filters, and the relative activity of filtrate and residue separately examined. To test the activity of these different substances, they were injected, hypodermically, into rabbits; the temperature was measured every two hours, and the dead body examined. The regular intermission of the fever and the swelling of the spleen and want of other changes, were employed as guides and measurements.

The results may be briefly summarized as follows:

1. The malarial poison is found in large quantities and is largely disseminated through the soil of malarial districts at a season when people are not yet attacked by disease.

2. At these times it may also be obtained, in especially favorable places, from the strata of air nearest the surface. To test this, 300 litres of air were thrown with great force and velocity against a glass plate covered with glue solution, to which the solid particles in the air adhered.

3. Stagnant water in malarial districts seemed not to contain the disease, although it may be, like the lake of Caprolace, extraordinarily rich in lower organisms. Their experiments indicate that a large quantity of water hinders the development of malarial poison and renders the germs which are present inactive.

4. By infection with the above fluids—some directly from the soil and others prepared by cultivation and filtration—a fever was produced in the animal, of the regular type, with intermissions, which lasted up to sixty hours, and an increase of temperature up to 40° C. (104° F.).

5. The filtered liquids caused but very slight increase of temperature, even when five times the quantity was injected. Even filtering through a double paper filter seems to remove the malarial poison.

6. Animals infected with malarial liquids all showed a swelling of the spleen, and in many of them was found a black pigment.

7. The organisms which were the real cause of the malaria, belong to the genus *Bacillus*. They are present in the soil of malarial regions, in the form of numerous movable brilliant spores of long oval shape, with a greater diameter of 0.95 micrometer. They grow, both in animals and in cultivating apparatus, into long threads, which are at first homogeneous, but afterward divide and develop again within the limbs. These spores first form on the walls, but finally the whole interior of the member becomes filled with these little bodies. Owing to their peculiar morphological action, they must be looked on as a new kind of bacilli, and have been named *Bacillus malariæ*.

8. These organisms will not develop if atmospheric oxygen is excluded, and hence belong to the class of Aerobii. They do not develop in water, but will in nitrogenous liquids, like solutions of glue, albumen and the fluids of the body. Sometimes the fibres reach the length of 0.06 to 0.084 mm.—*Scientific American*.

